# Underutilized plants increase biodiversity, improve food and nutrition security, reduce malnutrition, and enhance human health and well-being. Let’s put them back on the plate!

**DOI:** 10.1093/nutrit/nuad103

**Published:** 2023-08-29

**Authors:** Marija Knez, Marija Ranić, Mirjana Gurinović

**Affiliations:** Centre of Research Excellence in Nutrition and Metabolism, Institute for Medical Research, National Institute of Republic of Serbia, University of Belgrade, Belgrade, Serbia; Capacity Development Network in Nutrition in Central and Eastern Europe, Belgrade, Serbia; Centre of Research Excellence in Nutrition and Metabolism, Institute for Medical Research, National Institute of Republic of Serbia, University of Belgrade, Belgrade, Serbia; Capacity Development Network in Nutrition in Central and Eastern Europe, Belgrade, Serbia; Capacity Development Network in Nutrition in Central and Eastern Europe, Belgrade, Serbia

**Keywords:** biodiversity, food systems, forgotten foods, health benefits, noncommunicable diseases, nutrition security, underutilized plants

## Abstract

The global food system depends on a limited number of plant species. Plants with unsatisfactory nutritional value are overproduced, whereas the wide variety of nutrient-rich plant species used in earlier times remains neglected. Basing our diet on a few crops has wide-ranging negative consequences on nutrition and food security. Although still under-researched, underutilized plants are slowly starting to receive increased recognition. These plants have superior nutritional content and immense potential to contribute to food and nutrition security and increased sustainability. This narrative review provides evidence to encourage the promotion, domestication, and commercialization of underutilized plants. The anti-inflammatory, antidiabetic, and anticancer effects of some of underutilized plants are presented in this review. The outstanding ability of forgotten plants to increase food and nutrition security, boost dietary diversity, reduce malnutrition, and enhance human health and well-being is demonstrated. The main barriers and obstacles to reintroducing underutilized foods are reviewed and recommendations for overcoming nutrition and dietary-related challenges for re-establishing underutilized plants into the global food system are presented. The expansion of underutilized plants for human use is of paramount importance. The exceptional nutritional properties, bioactive potential, and proven health benefits of underutilized plants indicate that increased promotion, domestication, and commercialization of these plants should be strongly supported. Besides health benefits, marginalized plants have the potential to enhance human well-being and improve people’s lives in many ways, retain biodiversity, and develop local economies. Therefore, underutilized plants should be used in the broader context of well-balanced and healthy diets.

## INTRODUCTION

Sustainable food systems provide food security and nutrition for all in a way that social, environmental, and economic sustainability is not compromised for future generations.^1^ The sustainability of agricultural and food systems is threatened by several worldwide trends, the most important being an expected increase in global population. The world’s population is rapidly growing and expected to expand from the current 7.7 billion to close to 10 billion people by 2050, an increase of more than 30%.^2^ This will certainly lead to agricultural and food production expansion posing serious environmental concerns due to increased greenhouse gas emissions, increased deforestation, and water use, contributing further to ecological inadequacy and climate change.^3^ Environmental variations and fertilization are among the most important environmental factors influencing the chemical composition of plants and, therefore, the nutritional properties of harvested products.^4^

Furthermore, the main objective of the United Nations Decade of Action on Nutrition is “to eliminate malnutrition in all its forms, and to develop sustainable, resilient food systems for healthy diets” in line with the framework established at the Second International Conference on Nutrition in 2014.^5^ The concept proposes that the food safety and sustainability aspects should be integrated. More sustainable production of conventionally used plant species and reintroduction of marginalized crops should ensure both increased sustainability of food systems and biodiversity. More biodiverse agricultural and food systems are needed to feed all people by 2050 with healthy and nutritious food and at the same time ensure sustainability while protecting the environment.^6^

Overall, there is a need to create food production systems that maintain and stimulate diversity both within and between different plants, encouraging varietal and plant diversity.^7^^,^^8^ The global food basket should be expanded by reintroducing marginalized traditional plants. There is an increasing interest in introducing forgotten plants and creating foods and products that could contribute to human health and nutrition in innovative ways.^9^^,^^10^

In recent years, an increased amount of research has been devoted to underutilized foods, both in terms of identifying the geographical areas most affected by the elimination of certain forgotten, beneficial species and in terms of the cultivation, promotion, consumption, and health benefits of individual plants of interest. Our main objective for this review is to summarize the existing evidence on the nutritional and health benefits of underutilized plants, outline the main nutritional and dietary challenges to reintroducing underutilized foods, and propose strategies to address them.

## METHODS

The Web of Science and Scopus databases were searched using the keywords “underutilised,” “plants,” “health,” and “health benefits.” Of the 350 research articles and 112 review papers identified, only the most relevant to the topic were considered to provide appropriate data for a narrative review. We focused mainly on papers published in the past decade.

The information in this review is presented in multiple sections. The first 2 describe the current state of the global food system and nutrition crisis. Health benefits of certain underutilized plants are discussed. For this purpose, buckwheat, sowthistle, Armenian cucumber, and some neglected but more nutritionally valuable varieties of certain species such as tomato, grass pea, eggplant, and lentils are used as examples to demonstrate the health-promoting properties of various forgotten plants. The 7 plants were chosen to represent underutilized species because they meet some of the following criteria: water-energy efficiency; have high nutritional value; they are antioxidant rich, protein rich, and gluten free; have high potential to enter the food value chain; and have environmental resistance. The selected plants of interest represent various categories: cereals, grain legumes, fruits, vegetables, and wild crops. For example, buckwheat is an example of a cereal crop that is grown in smaller amounts in certain countries, but the general findings regarding lower production and health benefits of this crop could be applied to all other underutilized crops in the cereal category (eg, millet, quinoa, sorghum). The same is true for all other species mentioned.

### Current state of our global food system

Of 30 000 edible plant species identified, only 7000 have been used throughout history to meet food requirements.^11^ Currently, no more than 150 plant species are commercially cultured, with just 103 providing 90% of calories in the human diet.^12^

Food diversity has been lost in many countries worldwide. At present, the global food system relies on a limited variety of plant species. Four staple crops (wheat, rice, maize, and potato) represent more than 60% of the human energy supply.^13^

Plants with inadequate nutritional value are overproduced, whereas a wide variety of nutrient-rich plant species used in ancient times continues to be ignored. Improving the yield and, to a certain degree, nutritional content of a few major plants, have been the most important effort of the Green Revolution that took place from the 1960s to the 1980s. But, as it has been shown over the years, building our diet on a few crops only had wide-ranging negative outcomes on nutrition and food security.^14^ Increased crop yield was the main goal to ensure adequate caloric intake for an increasing number of people globally while less attention was devoted to the nutritional value of foods.

Increases in the population resulted in increased demands for food, and nutritional quality was inadvertently sacrificed while enough food was produced.^15^ Local crop diversity, culture, knowledge, and traditions have been neglected by decades of policies inclined to promote and support a few of the major crops only.^16^^,^^17^ Consequently, in many parts of the world, diets deficient in essential micronutrients and vitamins persist.^18^^,^^19^ The imbalances within the food system were not eradicated with a sufficient level of food production, even though enough food is produced for the population worldwide.^20^

Expected population growth suggests an increase in demand such that a doubling of food production may be required by 2050.^20^ However, unsustainable resources and planetary boundaries do not support further expansion of the current food system concentrated solely on increased yields and based merely on production intensification pathways.

The food system should develop in such a way as to offer sustained access to diversified and nutritious food to all. Alternative pathways and a transformation of the food system in a direction that fully meets people’s needs are required. Innovations in the food system are necessary, and new practices and food processing techniques are required to successfully move in the direction of creating a resilient and sustainable food system that ensures food security and provides sufficient supplies of adequate nutrition for the ever-increasing world population. As stated in a declaration of the World Food Summit in Rome in 1996, food security will be achieved when “all people, at all times, have physical and economic access to sufficient, safe, and nutritious food to meet their dietary needs and food preferences for an active and healthy life.”^21^

### Nutrition crisis

The Global Report on Food Crises states that 193 million people are currently living in severely food-insecure contexts.^22^ In 2020, 720 million to 811 million people encountered hunger and around 660 million people may still encounter hunger in 2030.^23^ The prevalence of undernourishment increased from 8.4% in 2019 to 9.9% in 2020. Almost 1 in 3 people did not have access to adequate food in 2020.^23^ Moderate or severe food uncertainty has been increasing slowly for 6 years and now affects more than 30% of the world’s population.^23^

Malnutrition is a growing problem in developing and developed countries. Although there is no official definition for this condition, it is described as the state of poor nourishment, insufficient and unbalanced absorption of macro- and micronutrients caused primarily by prolonged intake of inadequate diets, either under- or overnutrition.^24^^,^^25^ Malnutrition is both a cause and effect of poor health, it presents a risk factor for several diseases and, at the same time, it contributes to health deterioration.^18^^,^^26^

As projected by UNICEF, 47 million children younger than 5 years are suffering from wasting globally, 14 million of whom experienced severe wasting that threatens growth, development, and survival.^22^ The most recent data demonstrate that more than half of preschool-aged children and two-thirds of nonpregnant women of reproductive age worldwide suffer from micronutrient deficiencies. As reported, the estimated prevalence of deficiency in at least 1 of 3 core micronutrients (zinc, iron, and vitamin A) was 69% among nonpregnant women of reproductive age and 56% among preschool-aged children.^19^ Even though the prevalence was the highest in low-income and middle-income countries, approximately half of women and children in high-income populations were projected to have at least 1 micronutrient deficiency.^19^^,^^27^ Iron deficiency alone is prevalent in 1 in 5 women in the United Kingdon and the United States.^19^ Previous projections of 2 billion people being affected are now seen as underestimates; it is projected that close to 4 million people have 1 or more micronutrient deficiencies.^19^

On the other hand, there is the “double burden” of malnutrition. Obesity and overweight are found in developed and developing countries, and obesity rates expand rapidly even in regions where hunger exists. Approximately 2.5 times as many people are overweight as are undernourished, with a parallel increase in the number of overweight and obese people.^20^

Urbanization and globalization of the food system, social changes, market growth, and technological developments contributed to dietary changes, so now people are generally eating more food, but also more energy, protein, and fat-rich foods, than before. Even though individual diets are diversified to a degree, globally, diets have become even more homogenous, being based on a restricted number of foods produced.^28^

Although there were noticeable increases in the consumption of healthy, higher-quality foods, such as fruits, vegetables, and seeds, increased consumption of lower-quality foods was also evident. The intake of meat, sugar-sweetened beverages, and ultraprocessed foods significantly increased in the period from 1961 to 2014.^29^ As reported in the 2020 State of the Food Security and Nutrition in the World, the supply of cereals and pulses is the highest in low- and middle-income countries, whereas meat, fish, oil, and sugar are more readily available in middle- to higher-income countries.^30^ According to the World Health Organization, recommendations of 400 g/d for fruit and vegetables and 100–232 g/d for whole grains are far from being reached across all food systems.^30^

Briefly, shifts in dietary patterns and increased consumption of unhealthy foods with more sugar, salt, and fat are more prominent these days, as is the tendency toward overeating and reduced physical activity.^31^ The primary driver of food insecurity is poverty rather than lack of food availability.^32^^,^^33^ Then again, sufficient calorie intake does not necessarily ensure adequate nutritional value of food consumed. Nutrient deficiencies, such as lack of zinc, iron, vitamin A, and vitamin D, are present in both under- and overnourished individuals. Being overweight or obese increases the risk of developing noncommunicable diseases (eg, type 2 diabetes, cancers, and cardiovascular diseases [CVDs]). At least 2.7 million people globally die every year as a result of being overweight or obese.^34^ Ironically, although the prevalence of overweight and obesity has increased in all regions and in all countries around the world, the percentage of the population overacquiring food has increased even more dramatically in developing nations.^20^

In summary, the global food system is still far from reaching the long-standing sustainable development goal of eliminating hunger. Currently, access to safe, nutritious, and sufficient food for all people throughout the year is not available. Different forms of malnutrition coupled with a double-deficiency burden persevere. The COVID-19 pandemic made the situation even more challenging.^23^ Conflict, climate changes, growing inequalities, and economic slowdowns and declines are the main factors contributing to undermining food security.^23^

Production of sufficient quantities of food per se is not the only factor. The type of food produced, nutrient density, macro- and micronutrient content of foods, bioavailability, biodiversity, and sustainability can significantly determine the amount of food that should be produced to satisfy global food demand.

### Underutilized crops: the promising future foods?

The group of plant species no longer cultivated and consumed by humans is described by the following terms: orphan, underutilized, neglected, niche, minor, lesser known, and promising. These crops originated elsewhere but, for different reasons, became indigenized over decades of cultivation.^35^ The noncommodity crops, mainly wild or semi-domesticated varieties, are possibly implemented as minor crops in some countries while completely neglected in others. For long periods, most of these crops were overlooked by farmers, researchers, food producers, traders, consumers, and policymakers due to various genetic, agronomic, social, cultural, and economic reasons. Certain crop varieties got lost even before they got the opportunity to be researched, characterized, cultivated, and promoted, together with a wealth of knowledge on their cultivation and use.^36^

Now, although still under-researched, underutilized plants are gradually starting to obtain increased recognition due to their potential to diminish the risk of agricultural production systems and preserve the health of ecosystems.^36^ These plants have superior nutritional content and immense potential to contribute to food and nutritional security and reduce malnutrition.^37^ It is well known that increased consumption of locally accessible indigenous or traditional grains, roots, tubers, fruits, and vegetables improves nutrition and increases human productivity.^38^ Finally, neglected plants can empower the poor and marginalized, improve people’s diets, and promote cultural diversity.^36^

Underutilized crops have important nutritional and functional values, presenting sustainable sources of proteins, carbohydrates, essential fatty acids, vitamins, minerals, phytochemicals, and dietary fiber. Antioxidant, anti-inflammatory, and anticarcinogenic properties additionally add to the importance of these crops.^39^ Furthermore, underutilized crops provide essential micronutrients, complement staple crops, strengthen local gastronomic traditions, and could play a major role in improving the micronutrient content of the diets of millions of people worldwide. Their nutritional composition classifies them as beneficial food sources with the potential to reduce the risk of developing several noncommunicable diseases.

Finally, traditional food systems based on marginalized crops provide benefits not only in terms of physical health; they ensure the continuity of cultures, prevent loss of food heritage, and preserve a connection to local land, traditional cultures, and knowledge of local plants, in terms of cultivation, storage, and food preparation methods. For these reasons, an increased production and consumption of underutilized crops should be strongly encouraged. Better integration of wide variety of underutilized crops in agriculture and food systems is needed to ensure greater food diversity and increased environmental sustainability, food security, and reduced hidden hunger. An increased awareness of the importance of food composition in human health with an intensified consumers’ interest in nutritious plant-based foods allows room for such changes to occur.

### Health benefits of underutilized plants

Humans have always included wild plant species in their diets. These plants contain a pool of nutritional and medicinal compounds with remarkable pharmacological properties and health-promoting benefits.^40^ The nutritional potential of these foods should be kept and promoted and could play a very important role in health promotion and food diversification.^41^^,^^42^ Increased awareness of healthy food opens the space for the potential reintroduction of underutilized plants.^36^

**Figure 1:**
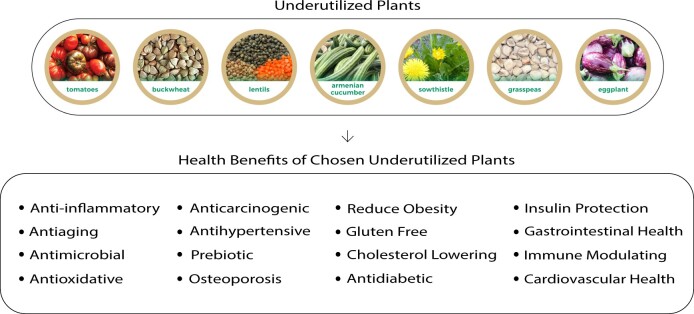
**Potential health benefits of underutilized plants**. Buckwheat, sowthistle, Armenian cucumber, and some neglected, but more nutritionally valuable, varieties of certain species, such as tomato, grass pea, eggplant, and lentils, are provided as examples

The cultivation, promotion, and implementation of underutilized crops can help reduce hunger and malnutrition, assist in fighting poverty, and ensure food security for many people worldwide.^43^^,^^44^ Many so-called wild underutilized plant species are considered food medicine due to the presence of several active compounds.^45^^,^^46^ Underutilized plants contain various nutritional components that, independently or in combination, demonstrate numerous health benefits, such as having anticancerous, antidiabetic, and cholesterol-lowering effects (Figure 1).

#### Forgotten plants and cardiovascular diseases

CVDs are the leading cause of death in many industrialized countries.^47^ Regular consumption of certain underutilized crops has been shown to reduce serum cholesterol levels and lower the risk of developing CVDs, stroke, and ischemic heart diseases. As shown, the consumption of buckwheat and buckwheat-related products reduced serum cholesterol concentrations in consumers by up to 13%, triglycerides by 5%, and blood glucose levels by 14%.^48^ The addition of buckwheat proteins to diets decreased cholesterol levels in the serum, gall bladder, and liver of consumers.^49^ Similarly, a tomato-rich diet is associated with a decrease in the risk of developing CVDs. A high-lycopene diet reduced the risk of developing CVDs by 14%, stroke by 23%, and overall mortality by 37%.^50^ Several antioxidants found in tomatoes, including lycopene, β-carotene, and vitamin C, protect lipoproteins and vascular cells from oxidation and preclude the development of atherosclerosis.^51^ Antiaggregatory, anti-inflammatory, and antiplatelet properties of these compounds were also documented, suggesting that a diet rich in tomatoes reduces lipid levels, lowers blood pressure, and decreases the risk of developing stroke and ischemic heart disease.^50^^,^^52^^,^^53^

Anthocyanins, present in eggplant and lentils, tend to prevent obesity by increasing high-density lipoprotein cholesterol levels and lowering serum triglyceride and cholesterol levels.^54^ Similarly, the mixture of nutrients in the Mediterranean diet, including the phytochemicals found in fresh fruits and vegetables has a positive impact on cardiovascular health.^55^

Comparably, foods rich in natural dietary antioxidants may lessen and counteract the risk of developing oxidative stress–related diseases. Wild edible plants lower the occurrence of CVDs.^56^ Antiplatelet, anticoagulant, and antioxidant potential of certain components (ie, carotenoids and flavonoids) present in *Sonchus* (sowthistle) could be protective against CVDs.^57^ Likewise, lentils possess substances with cardioprotective effects, they can reduce the development of hypertension, suppress pathological vascular remodeling, and help in maintaining vascular function and protecting against cardiac hypertrophy.^58–60^ Finally, the active substance present in *Cucumis melo* shows cardioprotective roles.^61^

#### Anticancer potential of certain underutilized plants

As a group, cancers remain among the leading causes of death, accounting for more than 7 million cancer-associated deaths globally. Twelve million new cancer cases are identified annually.^62^

Lycopene and β-carotene, essential carotenoids found in tomatoes, have strong anticancer properties, with antioxidant, anti-inflammatory, angiogenic, and anti–lipid peroxidation activities.^63–65^ The health benefits of tomatoes are additionally enhanced when lycopene is dissolved in olive oil and heated.^66^ These substances protect against carcinogenesis by inducing apoptosis in cancer cells.^63^^,^^67^

Anticancer properties of lycopene in prostate and breast cancers have also been documented.^68^ Similarly, a significant inverse relationship between tomato consumption and the risk of prostate cancer has been reported.^69^ The inhibitory effects of lycopene on the signaling pathway in human colon cancer cells was shown long ago.^70^ Interestingly, the angiogenic and anticancer potential of lycopene was higher in people who consumed tomato foods for longer periods.^71^

Tomatoes contain a variety of other chemoprotective and anticancer compounds, such as vitamin C, β-carotene, and ferulic acid, meaning that, most likely, strong anticancer properties of this fruit exist due to the combination of these compounds rather than lycopene alone.^72^^,^^73^ Similarly, decreased risk of developing various types of cancers was reported with higher consumption of plants with high phenolic acid and isoflavone content.^74–76^

A diet rich in natural antioxidants could prevent the development of endometrial and colorectal cancers.^77^ Underutilized crops contain a variety of antioxidants. For example, the skin of eggplant inhibits hydroxyl radical generation due to high superoxide-scavenging activity.^78^ Eggplant cells have a toxic effect on cancer cells, and phenolic compounds lead to the apoptosis of many human cancer cells, demonstrating anticancerogenic effects.^79^ In addition, the consumption of carotenoid-rich foods has been linked to a diminished risk of several types of cancers.^80^

Furthermore, lentils are high in fiber and have been shown to decrease systemic inflammation and reduce susceptibility for the development of certain cancers.^81^  *C. melo* contains high amounts of carotene with high antioxidant, anti-inflammatory, and anticancer activities.^82^^,^^83^ Consumption of *C. melo* led to the repression of several critical steps in tumor growth, disrupting angiogenesis and tumor progression.^84^

Finally, antitumor activities have been demonstrated with the consumption of plants containing taraxasterol and other sterol components.^85^ For instance, regular consumption of pseudocereals, such as buckwheat, has been linked to a reduced risk of developing certain types of cancers.^86^ The antioxidative ability of human blood serum increased with the consumption of honey from buckwheat flowers.^87^

#### Diabetes mellitus and underutilized plants

Diabetes is an epidemic disease that presents with elevated concentrations of blood glucose levels.^88^ Both type 1 diabetes (inability of the pancreas to produce insulin) and type 2 diabetes (more common, lack of ability to use available insulin) are related to the development of heart and kidney diseases, nerve damage, and blindness. Diets high in saturated fats and trans fatty acids, in addition to low physical activity and obesity, are the main contributing factors to the development of this ailment.^89^ Regular consumption of resistant starches, oligosaccharides, and dietary fiber (nutritional components of many underutilized crops) contribute to low glycemic indices by slowing the digestion of starches in the small intestine and delaying the gastric-emptying rate. The presence of phytic acid, amylose inhibitors, lectins, and phenolic compounds is directly linked to reduced blood glucose and insulin levels.^90^ A reverse correlation was reported between plasma β-carotene, lycopene, and glucose intolerance.^91^ Lycopene intake correlated negatively with fasting blood glucose and glycated hemoglobin levels in patients with type 2 diabetes.^92^

A decrease in diabetes-induced hyperglycemia, dyslipidemia, and oxidative stress with the consumption of lycopene- and tomato-enriched diets was recently documented in detail.^93^ Lycopene protected women against gestational diabetes–associated hyperglycemia and indicated that regular consumption can offer a protective effect.^94^

Tomatoes contain the glycoalkaloid esculeoside A, which is recommended as a functional supplement for diabetes.^95^ Tomato compounds have beneficial effects on diabetes.^96^ Collins et al,^65^ in their recently published review, point out that tomato or lycopene-rich diets are linked to a diverse range of health benefits. Regular consumption of tomatoes reduces the risk of developing cardiovascular, bowel, neurodegenerative diseases, and cancers, improves skin health, and stimulates the immune response.^65^ Besides the lycopene, tomatoes contain many other bioactive components (ie, polyphenols and phytosterols) with valuable anticancer, skin, and cardiovascular health properties.^65^

Likewise, caffeic acid present in *Sonchus* and tomatoes stimulate insulin secretion from pancreatic cells and lowers blood sugar levels.^6^ A heterogenous mix of components (flavonoids, phenolic acid, and phytosterols) found in examined underutilized plants demonstrate antidiabetic properties.^97^

Glucolipid metabolism is improved in the presence of fructans, a naturally occurring component of *Sonchus*. *Sonchus* species also have antidiabetic and antioxidant properties.^98^ Similarly, antidiabetic properties of *C. melo* exhibited via the inhibition of certain enzymes contributed to slower glucose absorption in the small intestine and accomplished better postprandial blood glucose control.^99^

Similarly, lentils have been shown helpful for the management of risk factors linked to hypertension, obesity, insulin resistance, dyslipidemia, and CVDs.^81^ Lentils lower blood glucose levels and improve high-density lipoprotein cholesterol in people with diabetes.^100^ Cooked lentils were more effective in lowering blood glucose levels, with no differences observed between the effect of whole compared with dehulled lentils.^100^

Likewise, buckwheat is an excellent crop for controlling blood sugar levels because it lowers the glycemic index. Patients consuming buckwheat products had lower insulin and plasma glucose levels and higher postprandial satiety in comparison with patients eating white bread.^101^^,^^102^

Finally, eggplant’s high fiber content and low soluble-carbohydrate levels make this plant a good choice for managing type 2 diabetes.^103^ Likewise, anthocyanins present in eggplant appear to help control diabetes.^104^ Eggplant-rich diets have the potential to reduce hypertension, hyperlipidemia, and oxidative stress in people with diabetes.^103^

### Marginalized plants can prevent nutrition-related health problems throughout the lifespan

**Figure 2:**
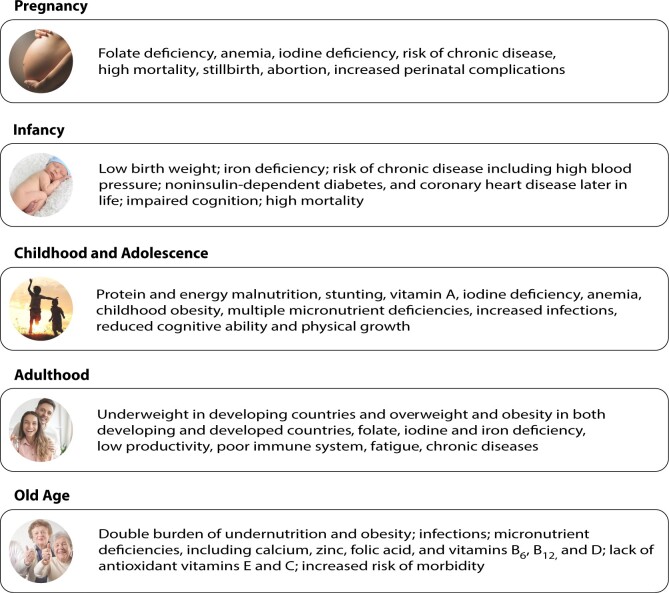
**Nutritional and health problems in each of the lifespan stages, from pregnancy to old age, that could be conquered with the regular consumption of underutilized plants**. Figure created based on information from Bernstein and McMahon,^107^ Ahmed,^108^ and Merrick.^109^

Underutilized crops have great potential for improving the overall nutritional status through the lifespan because these foods provide health benefits beyond those attributable to traditional nutrients. Figure 2 describes nutritional and health problems in each of the lifespan stages, from pregnancy to old age, that could be alleviated and/or prevented with regular consumption of underutilized plants.

Underutilized plants or foods containing marginalized plant constituents appear to have great potential and scope for alleviating and/or preventing malnutrition at different stages of life.^43^^,^^105^^,^^106^ As discussed earlier in this article, these foods could play an important role in reducing the risk of chronic diseases in children, adults, and the elderly, thus helping maintain a healthy lifestyle.^107–111^

### Underutilized plants improve food and nutrition security and enhance human well-being

Food is our daily requirement and a vehicle of our family traditions, cultural values, and personal beliefs. Diet is 1 of the key determinants of our health status. It influences nutritional status and has a main role in preventing several different diseases and disorders. The dietary habits of people are modulated by available information on the benefits of responsible food choices, lifetime preferences, and several different internal and external factors.^112^ Very often, culture is the main element that determines food choices, the way the food is produced, prepared, and eaten.^113^ In addition, globalization is also influencing traditional food choices and eating habits.^114^

The conception of healthiness varies across countries and geographical regions, but generally, there has been increasing attention to the role of diet in human health in recent years. People are becoming more and more aware of the importance of a healthy lifestyle. People’s awareness of food quality, personalized foods, and nutrition has increased,^115^ enabling the development of alternative solutions for healthier plant-based diets. Additionally, there is a global demand to enhance diet quality and diminish inequities in access to affordable, nutritious, and sustainably produced foods in all countries across the globe.^116^

Plants have provided for humanity for thousands of years and are important sources of specific nutritional components with various prohealth activities and beneficial health properties. Healing properties are attributable to the existence of various nutrients, vitamins, minerals, bioactive components, fatty acids, and a wide variety of other substances. The popularity and current intention of consumers to include more plant-based foods in their daily diets^117^ provide room for the inclusion of forgotten and marginalized but promising alternative plant sources. Underutilized plants offer opportunities to enhance diets with healthier food in ways that reflect food culture, besides contributing to making meals more interesting. Furthermore, underutilized plants represent enormous opportunities for combating hunger and malnutrition, improving biodiversity, and advancing agricultural and nutritional development.

Plant-heavy diets support biodiversity. The Mediterranean dietary pattern, mainly a plant-based diet, is recognized as a reliable and effective dietary pattern for environmental and economical sustainability.^118^ As such, changes within the food system are required. Underutilized plants and plant-based diets in general should be supported and promoted because of beneficial effects in terms of human health, nutrition, and biodiversity.

As an example, the addition of underutilized foods to diets helped reduce macro- and micronutrient deficiencies and iron deficiency in Kenyan women and children.^38^ Forgotten crops aided in combating food and nutrition insecurities and hidden hunger in Burkina Faso.^119^ Similarly, underutilized plants improved the life of people in African countries by keeping traditional knowledge, protecting the environment, and maintaining biodiversity. Regular consumption of 3 underutilized crops rich in vitamins and minerals, *Diospyros mespiliformis* (jackalberry), *Ziziphus mauritiania* (jujube), and *Balanites aegyptica* (desert date or soap berry tree) proved beneficial in ensuring food security and improving the nutrition status of consumers in Burkina Faso.^119^ Similarly, forgotten vegetables cultivated and eaten in rural Africa are shown to be rich in iron, zinc, and β-carotene.^120^ Ancient *Cleome gynandra*, the African spider flower plant; amaranth; African nightshade; and pumpkin seeds are full of valuable minerals and vitamins and have tremendous potential to improve nutritional security in these areas.^121^

Apart from the advantages to health, underutilized crops possess the capacity to enhance human welfare and positively impact individuals’ lives through various means. These plants offer supplementary food resources, safeguard the ecosystem, foster an understanding of locally accessible flora, guarantee the sustainable utilization of soil and water, conserve biodiversity, elevate quality of life, and foster the growth of local economies.^122–124^

**Figure 3:**
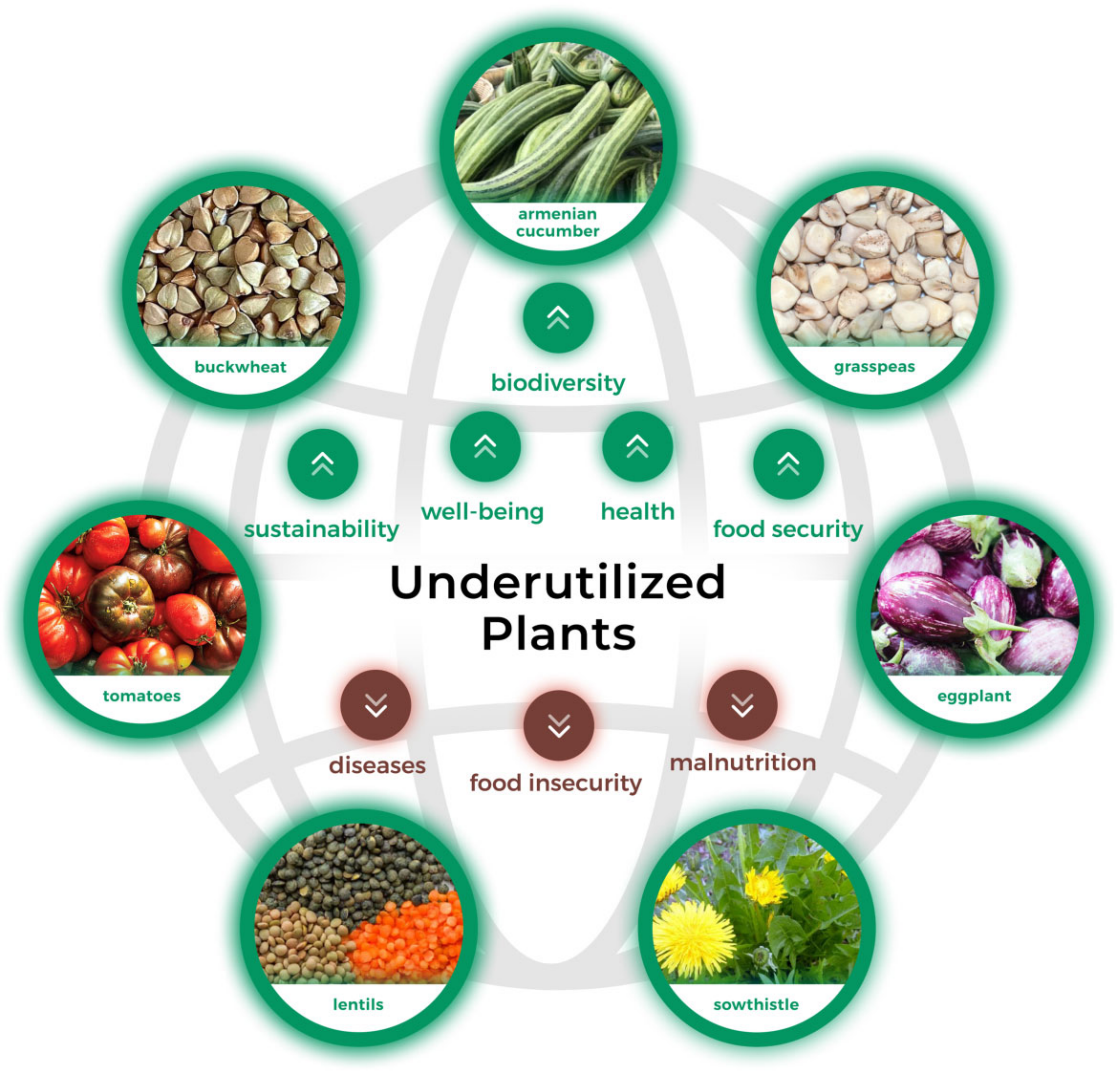
**The potential of underutilized plants to transform the global food system.** Buckwheat, sowthistle, Armenian cucumber, tomato, grass pea, eggplant, and lentils are offered as examples.

In summary, marginalized and forgotten plant species are nutrient rich and could help increase biodiversity, reduce malnutrition, improve human health and well-being, and contribute to global food security (Figure 3). The exceptional nutritional characteristics, bioactive potential, and demonstrated health benefits of underutilized plants prove that enhanced promotion, domestication, and commercialization of these plants are highly recommended. Selected marginalized plants should be considered and implemented in a broader context of balanced and healthy diets.

### The main challenges and obstacles for reintroduction of underutilized plants

Neglected and underutilized plants are often described as “non-commodity wild or cultivated plant species, including crop wild relatives, that were once popular but have since been neglected by mainstream agriculture due to a range of agronomic, genetic, economic, social, and cultural reasons.”^125^

Orphaned species were predominantly grown by resource-poor farmers, primarily women, who used the available seeds on small landholdings in certain agro-ecological niches and on marginal and submarginal lands to provide their families with food of high nutritional value.^126^ These plants were also used as animal feed and in other agricultural applications generating income for resource-poor farmers.^127^ However, because of their lack of economic value, many of these plants have been neglected by the international scientific community and industry compared with commodities such as rice, wheat, and maize.

An extensive monoculture, agricultural transformation, and an inclination toward more high-yielding varieties were the main reasons for the underutilization of certain beneficial plants.^128^ Demographic shifts complemented by dietary changes, the long preparation time, the advanced age of the people who knew how to prepare these foods, the limited supply of the forgotten foods, and lack of innovative postharvest and processing technologies are some of the factors contributing to the disappearance of these foods.^10^

Stigmatization, a negative image of “food of the poor,” was an additional factor that led to reduced production and consumption of these plants.^10^ Traditional and wild relatives of plants were often seen as old-fashioned, linked to the rural poor, especially in the eyes of recently urbanized populations in developed countries.^10^ In addition to other reasons, the forgotten plants remain forgotten because people are uncertain of how they can be used as food.^10^

Remarkably, underutilized crops have endured even without formal support, which implies that besides their exceptional nutritional value and beneficial effects on human health, they contain some desirable traits that could be useful for building resilience and adaptation to climate-changing environments.^129^ Therefore, the re-establishment of these plants to the global food system is desirable. To find solutions for overcoming the obstacles for the reintroduction of these crops and to bring the neglected species back to cultivation and use, a comprehensive understanding of potential reasons for the elimination of these plants is necessary. The main reasons for altered or eliminated consumption and cultivation of certain plants and the most important identified factors for the reintroduction of underutilized plants are listed in Table 1.^10^^,^^36^^,^^105^

**Table 1:** Reasons for altered or eliminated consumption and cultivation of certain plants and the most important factors for the reintroduction of underutilized plants

Problems with production and harvesting, yield, changes in land use
Absence of genome sequences for certain crops, lack of seeds availability and supply systems, low processing of seeds of certain varieties, and inadequate dissemination of materials and seeds
Biotic factors: insects, diseases, and weeds
Abiotic issues: temperature, soil fertility, waterlogging, drought
Presence of toxins and allergenic compounds and overuse of pesticides and fertilizers
Climate change and environmental pollution
Agronomical traits, germplasm collection, genetic factors, the limited number of species used as food, ecosystem degradation
Poor economic competitiveness of underutilized plants compared with staple crops, marketing constraints, short shelf life
Green revolution issues, self-incompatibility of certain plants
Inefficiency in producing, storing, and processing of these crops and their low commercial value
Disorganized or nonexistent food supply chains and a lack of market infrastructure
Expansion and cultivation of more common higher-yield cereal crops, monocultures
Increased cultivation of so-called exotic varieties
Changes in dietary patterns due to industrialization and migration of farm labor to urban regions
The lack of sound baseline knowledge and awareness of the nutritional and health-protective and health-promoting properties of local varieties
Loss of indigenous knowledge and absence of culinary skills for the preparation of foods based on these plants
Unaccustomed taste of these foods, nonpopular recipes
Negative associations with a poor rural way of life and low social status, negative cultural stereotypes against these traditional foods (eg, “this is what poor people eat”)
Lack of policy recommendations to support scientific research on underutilized crops
Political and economic reasons, lack of appropriate strategies, plans, national programs, and schemes for reintroduction, implementation, broader-level cultivation, and consumption of these plants.

### Recommendations for overcoming nutrition- and dietary-related challenges in reintroducing underutilized plants into the global food system

The introduction, maintenance, management, and promotion of underutilized plants present several challenges from different scientific fields. Although several barriers are presented that should not be neglected and separated from the other obstacles, we have focused in this article primarily on the nutritional challenges of reintroducing underutilized plants. Based on the information presented in the previous section, we propose certain ideas and actions to overcome these obstacles. These are presented in Table 2.

**Table 2:** Ideas and actions to overcome the nutritional challenges of reintroducing underutilized plants

Improve knowledge of available cultivars and nutritional value of local and traditional foods; build the capacity of stakeholders who would support nutrition and food scientists in their actions
Invest in research to obtain suitable and sufficient knowledge of dietary and health benefits of underutilized crops and determine their full potential for improving human nutrition in developed and developing countries
Combine scientific and indigenous knowledge of nutritional and health benefits of these crops; disseminate the knowledge among both the rural poor and urban consumers
Demonstrate, validate, and promote the nutritional and health benefits of underutilized crops at the national, regional, and international levels through information campaigns and media publications to encourage consumption of underutilized crops
Develop skills and capacity in nutrition development; stimulate cooperation with experts from different disciplines: health, agronomy, sociology, economics, and business
Raise awareness of the nutritional value of underutilized crops and strengthen the links between local farmers, food producers, consumers, local chefs, restaurants, and food retailers in terms of the production, marketing, and promotion of these plants and foods
Use communication and education campaigns to change peoples’ perception of underutilized crops; reorientate the widespread prejudice of “food of the poor,” educate people, and address existing negative connotations
Create programs for advertising underutilized foods of interest, encourage their use in everyday cooking, promote their use as both food and medicine, and stimulate improvements of culinary skills of consumers
Support food producers with necessary information related to nutritional benefits of underutilized plants to ensure better availability of these foods and related products on markets
Diversify the ways in which the underutilized plants are used by creating and promoting new food dishes and novel food products containing these plants; propose the introduction of underutilized plants in organized feeding programs (ie, school canteens)
Help farmers and producers increase knowledge of and willingness to cultivate and produce plants of interest by providing information on the nutritional benefits and human well-being
Provide policymakers with evidence (ie, successful, small-scale, real-life stories showing how underutilized plants benefit communities and have the potential to contribute greatly to the nutritional, health status, and overall well-being of consumers)
Reduce political and economic neglect of underutilized species; develop national nutritional policies to support the promotion and application of these foods and related products on local and national levels; encourage cooperation at an international level

## CONCLUSION

With the presence of a sedentary lifestyle, obesity, global food insecurity, and malnutrition, it becomes of utmost importance to encourage increased consumption of underutilized plants in the diet to take advantage of their unique nutritional and health-promoting characteristics that could prevent several diseases of modern life. Western-style diets high in saturated fats, salt, sugar, and processed foods replaced traditional diets based on local, nutrient-rich, and diverse foods, thus increasing the incidence of noncommunicable diseases. Nutritional deficiencies are more often related to undernourishment, but it is also possible to consume sufficient calories and still have deficiencies in important micronutrients.

In this sense, underutilized crops can help in fighting hidden hunger while enhancing diets high in refined carbohydrates and fats. As demonstrated, marginalized plants are nutritionally and phytochemically dense and contain a set of antinutritional components, all with well-known beneficial effects on human health. Different nutritional components found in underutilized plants can serve as natural remedies for many diseases due to their hypolipidemic, antiobesity, anti-inflammatory, anticholinergic, hepatoprotective, antioxidant, anticancer, and antidiabetic effects.

The synergistic effects of compounds found in each of examined plants are expected to outweigh the benefits of individual constituents. The need for gluten-free diets could help accelerate the promotion and use of buckwheat and other pseudocereals with similar health benefits.

Consumption of underutilized crops and foods containing them is associated with a lower risk of developing major diseases in industrialized countries, such as diabetes, celiac disease, CVD, and various cancers. In addition, underutilized plants have the potential to increase plant and food diversity and improve human health and well-being. Similarly, regular consumption of underutilized plants could prevent and/or alleviate various nutritional or health problems at any stage of life.

Interventions based on underutilized plants could be cost-effective investments in the global development of secure and healthy food systems for all. Identification of barriers and challenges to reintroducing underutilized plants into the food system presented in this article is an important step toward offering applicable solutions for addressing nutrition-related challenges. Marginalized plants can address different goals and pillars of nutritional and food security and should be considered and used in a broader context of balanced and healthy diets. Finally, underutilized plants can be used for the preparation of a wide range of innovative food dishes and food products that could fulfil the need for healthier and more biodiverse food systems.
